# Expanding the usage of cryoablation as focal therapy for prostate tumour near the rectum

**DOI:** 10.1002/bco2.283

**Published:** 2023-12-27

**Authors:** Yiu Chung Lam, Chi Man Ng

**Affiliations:** ^1^ Division of Urology, Department of Surgery Princess Margaret Hospital Hong Kong SAR China

**Keywords:** cryoablation, cryotherapy, prostate cancer, rectum, spacer

Since radical treatment yields no major advantages in terms of prostate cancer specific and overall survival comparing with expectant management,^1^ focal therapy (FT) is becoming more popular, providing a balance of oncological control and functional outcomes.^2^ Cryotherapy is one of the most commonly use focal treatment for intermediate risk localized prostate cancer. It involves freezing and thawing the target areas of the prostate and ultimately inducing an extremely cold temperature within the prostate tumour. It results in cell death by tissue ablation. At the same time, it is mandatory to keep a certain distance between the rectal wall and the prostate ablative area during cryoablation for not only protecting the rectal wall from thermal injury but also allowing a more powerful ablative energy output. So prostate cancer in peripheral zone near the rectum is relatively contraindicated for cryotherapy.

In order to expand the usage of cryotherapy in prostate cancer near the rectum, different innovative methods have been used including saline displacement of the rectal wall,^3^ proactive rectal warming using cryoprobe^4^ and Hydrogel spacer (absorbable gel) injection into the Denovilliers' space to widen the distance between the rectum and the prostate.^5^ Barrigel® is a new rectal spacer that can be sculpted in place to create a custom fit to the anatomy for minimizing the complications associated with radiation therapy in prostate cancer treatment. It is made from Non‐Animal Stabilized Hyaluronic Acid (NASHA®) and is fully absorbable by the body. It is used in prostate cancer radiotherapy but not in ablative therapy, to the best of our knowledge. Therefore, our study aimed at assessing the feasibility and safety of using SpaceOAR™ Hydrogel and Barrigel® as a spacer between the rectum and the prostate in cryoablation for prostate cancer near the rectum.

We conducted the experimental study in operating theatre. A porcine model of pork belly size 20 × 20 × 10 cm was used for the study. Nonetheless, porcine model had been used in transurethral and percutaneous bladder cryoablation experimental studies.^6^, ^7^ Under ultrasound (USG) guidance (High resolution 3D side fire USG probe, KOELIS Trinity®), 10 cc of saline was injected into the fascial plane of muscles as hydrodissection. Then two injections (5 cc per injection) of SpaceOAR™ Hydrogel were carried out to create a 10 mm thickness space between two muscle layers. A cryoprobe (14G, IcePearl™ 2.1CX straight, Boston Scientific) was placed at a target point 10 mm superior to upper margin of the SpaceOAR™ Hydrogel. The IcePearl™ cryoprobe can form iceball with −40°C isothermal area 10 mm distant from the distal portion of the needle. Then two temperature sensors were placed at superior (TS1) and inferior (TS2) margin of the SpaceOAR™ Hydrogel (Figure 1). TS1 was used to measure the temperature of the edge of iceball, and the temperature difference between the two sensors could demonstrate the insulation property of the spacer. Two freeze–thaw cycles (ICEFX™ Cryoablation system, Boston Scientific) were used to expand the iceball under real‐time USG monitoring. The speed of the iceball formation, its configuration, and any change of the shape of the spacer were closely observed. The entire procedure time was 40 min. The temperature at each thermocouples was recorded continuously during the whole procedure. After the end of second freeze–thaw cycle, the porcine model was cut along the line of cryoprobe insertion to take a closer look of the macroscopic feature of the spacer and surrounding tissue.

**FIGURE 1:**
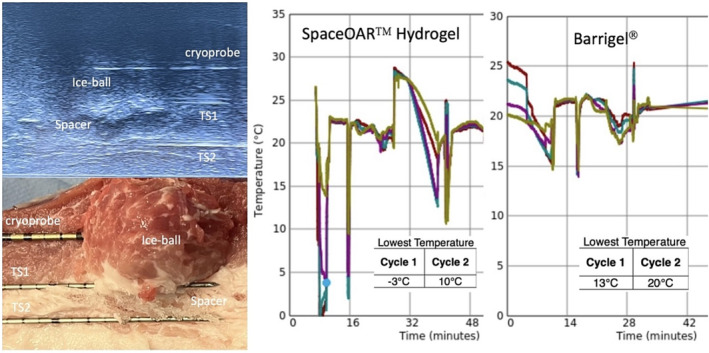
Left upper: An ultrasound image of the ice ball, spacer, cryoprobe and thermocouples (TS1/TS2). Left lower: The cut open view of the model after the second freeze–flaw cycle. Right side: Temperature change during the experiment: cycle 1 TS1, cycle 2 TS2, cycle 2 TS1 and cycle 2 TS2.

Thereafter, the procedure repeated by using three injections (3 cc per injection) of Barrigel® as the spacer, without saline hydrodissection, in identical investigation setting. The lowest temperature recorded by TS1 for the testing either using SpaceOAR™ Hydrogel or Barrigel® as spacer were both −40°C. Meanwhile, the lowest temperature recorded by TS2 was −3°C and 13°C for the SpaceOAR™ Hydrogel and Barrigel®, respectively, in the two freeze–thaw cycles (Figure 1). The created mass for both SpaceOAR™ Hydrogel and Barrigel® were only slightly alternated in shape by iceball compression during the freeze cycle. There was no alternation of macroscopic appearance and infiltration of surrounding tissue for both spacers after the whole procedure (Figure 1).

Nowadays, urologists were growing interest in using FT in localized prostate cancer treatment. An ‘À la Carte’ approach using different energy modalities for FT according to intraprostatic tumour location was advocated.^8^ High‐intensity focused ultrasound (HIFU) was favourable for posterior tumour because of the shorter focal distance, more precise contouring of the target area and, most importantly, the least danger to the rectum in proximity. However, not every urology centre had all FT options for prostate cancer due to limitation of resources. A recently published study on cryoablation of prostate tumour without the use of rectal spacer showed men with posterior tumours had higher chance of having clinically significant prostate cancer (csPCa) (21/48, 43.7%) than men with anterior tumours (4/23, 17.4%) on 18 months Magnetic Resonance Guided prostate Biopsy (MRGB).^9^ Therefore, when cryoablation was the only energy modality available, it was encouraging to use rectal spacer as an adjunct in cryoablative treatment for posterior prostate tumour to minimize rectal thermal injury, while assuring adequate treatment to the lesions. To date, there is only one study showing the feasibility of using rectal spacer, the SpaceOAR™ Hydrogel, in cryoablation of prostate tumour near the rectum.^5^ Our study showed that using Barrigel® to expand the Denovilliers' space is another safe and promising option.

During the entire process of investigation, the lowest temperature measured in model using Barrigel® was much higher than SpaceOAR™ Hydrogel (13°C vs. −3°C). This finding might be due to different physical and chemical properties of the spacers. To begin with, SpaceOAR™ Hydrogel consisted of mostly water (90%) and polyethylene glycol (PEG) that would be fixed into certain shape within 10 s after injection. It required pre‐injection saline hydrodissection to create space in the target area. As a result, good injection technique was crucial during implantation in order to turn the gel‐like liquid state hydrogel into an even thickness waxy solid mass in seconds. So it was not surprising that some parts of the hydrogel mass were not thick enough to block the ‘blizzard’ effectively. In contrast, Barrigel® was made from Non‐Animal Stabilized Hyaluronic Acid (NASHA®), which was similar to the hyaluronic acid naturally present in the human body. Its internal structural engineering allowed for both precise placement and lifting power. At the same time, the jelly‐like physical property of Barrigel® was more flexible in creating any shape of implant to achieve optimal spacing effect. In De Castro Abreu et al.'s study, they used four injections (total 20 cc) of hydrogel to achieve satisfactory spacing effect,^5^ so cost effectiveness (20 cc SpaceOAR™ Hydrogel: HKD$24000 vs. 9 cc Barrigel®: HKD$13000) was a concern.

Moreover, the lowest temperature of TS2 detected in hydrogel of around 0°C could be explained by pre‐injection saline hydrodissection and high water content of hydrogel. Since water would condense into ice once it reached 0°C, ice crystal might form in the hydrogel spacer when it touched on the expanding iceball. So the ambient temperature of the hydrogel spacer might be zero or below. Therefore, SpaceOAR™ Hydrogel might not be an effectual cold insulating agent as compared to Barrigel® in which the main component was hyaluronic acid.

Increasing the distance between the prostate and the rectal wall by injection of spacer into the Denovilliers' space during cryoablation for posterior prostate tumour is a feasible way to enhance the safety of the procedure. The water‐rich gels may also provide additional insulating effect. Temperature mapping and macroscopic appearance of both spacers showed safety of this technique.

## AUTHOR CONTRIBUTIONS

**Yiu Chung Lam:** Conceptualization (lead); data curation (lead); formal analysis (lead); investigation (equal); methodology (lead); project administration (lead); resources (lead); visualization (lead); writing—original draft (lead); writing—review and editing (equal). **Chi Man Ng:** Conceptualization (supporting); data curation (supporting); formal analysis (supporting); investigation (equal); methodology (supporting); project administration (supporting); resources (supporting); visualization (supporting); writing—original draft (supporting); writing—review and editing (equal).

## CONFLICT OF INTEREST STATEMENT

SpaceOAR™ and ICEFX™ Cryoablation system used in the experiment were provided by Boston Scientific while Barrigel® was provided by Kerry Medical.
